# Obituary

**Published:** 1883-12

**Authors:** 


### Obituary.

At a meeting of the dentists in the city of Springfield, Ohio, held at the office of Dr. F. C. Runyon, Tuesday, October 30th, Dr. J. Ramsey was called to the chair.

The Chairman announced that the object of the meeting was to take some action in memory of Dr. M. M. Oldham, who died at New Orleans, Saturday, October 27th, 1883.

On motion, Drs. C. R. Converse, W. H. Smith, and T. A. Lewis were appointed a committee to draft resolutions.

The Committee on Resolutions submitted their report, which was, on motion, received and adopted, viz.:

God having called from among us our friend and brother, Dr. M. M. Oldham; while we with humble reverence submit to Him who doeth all things well, it is fitting that we assemble ourselves together to do honor to one who had, for so many years, honored our profession.

*Resolved*, That we deeply sympathize with his family and friends in their sore bereavement, and tender them our heartfelt condolence.

*Resolved*, That we attend the funeral of our dead brother in a body.

*Resolved*, That a copy of these resolutions be sent to the family of deceased, to the daily papers, and to the dental journals for publication.

